# Hematological and Biochemical Data Obtained in Rural Northern Uganda

**DOI:** 10.3390/ijerph110504870

**Published:** 2014-05-06

**Authors:** Nirianne M. Q. Palacpac, Edward Ntege, Betty Balikagala, Adoke Yeka, Hiroki Shirai, Nahoko Suzuki, Christopher Nsereko, Bernard N. Kanoi, Takuya Okada, Thomas G. Egwang, Toshihiro Horii

**Affiliations:** 1 Department of Molecular Protozoology, Research Institute for Microbial Diseases, Osaka University, Suita, Osaka 565-0871, Japan; E-Mail: nirian@biken.osaka-u.ac.jp; 2 Med Biotech Laboratories, P.O. Box 9364, Kampala, Uganda; E-Mails: ehntege@gmail.com (E.N.); bettymawagali@gmail.com (B.B.); yadoke@yahoo.com (A.Y.); bnkanoi@gmail.com (B.N.K.); tgegwang@gmail.com (T.G.E.); 3 Proteo-Science Center, Ehime University, 3 Bunkyo-cho, Matsuyama, Ehime 790-8577, Japan; 4 Makerere University School of Public Health, P.O. Box 7072, Kampala, Uganda; 5 The Research Foundation for Microbial Diseases of Osaka University, 2-9-41 Yahata-cho, Kanonji, Kagawa 768-0061, Japan; E-Mails: hshirai@mail.biken.or.jp (H.S.); napokoko@gmail.com (N.S.); tkokada@mail.biken.or.jp (T.O.); 6 The London School of Economics and Political Science, Houghton Street, London WC2A 2AE, UK; 7 Lira Medical Center, Lira, Uganda; E-Mail: chrisdoc23@yahoo.com; 8 Muhimbili University of Health and Allied Sciences, United Nations Road, Dar es Salaam, P.O. Box 65001, Tanzania

**Keywords:** reference intervals, clinical trials, hematology, biochemistry, adult reference values, children reference values

## Abstract

Reference intervals for common hematological and clinical chemistry parameters constitute an important basis for health care. Moreover, with increasing priority in drug and vaccine development for infectious diseases in Africa, the first priority is the safety evaluation and tolerability of the candidate interventions in healthy populations. To accurately assess health status and address adverse events, clinical reference intervals in the target population are necessary. We report on hematological and biochemical indices from healthy volunteers who participated in a clinical trial in Lira, northern Uganda. Median and nonparametric 95% percentiles on five hematology and 15 biochemistry analytes are shown. Although most hematological analytes conformed to reported reference intervals and trends in Africa, literature review from different African countries highlight the need for a region-specific children reference interval that can be appropriate for the population.

## 1. Introduction

Reference intervals are used to describe the dispersion or fluctuation of analytes in healthy individuals; as indicators of good health or for accurate interpretation of physiological or pathological conditions in clinical and research environments. They are of particular importance in clinical trials especially in Africa [1], guiding the screening and enrollment of prospective participants as well as the safety assessment of investigational products. Phase I/II trials rely on enrollment and assessment of healthy participants for proper safety evaluation of interventions prior to phase III trials where safety and efficacy are evaluated on a larger scale. In April–May and September–October 2010, screening for enrollment in a phase Ib age de-escalation vaccine trial to assess the safety and immunogenicity of the BK-SE36 malaria vaccine was conducted at Lira Medical Centre (LMC), Lira, Uganda [2]. The trial assessed for eligibility 100 adults (>21 year-old) and 150 volunteers aged 6–20 years. Inclusion and exclusion criteria were based on reference values obtained from LMC as well as from a number of publications that address reference intervals in Africa [3,4,5,6,7]. From our experience, the reference values were not easily applicable to the phase 1b clinical trial, one of the reasons that contributed to a high screening to enrollment ratio. This resulted in logistical, personnel, as well as financial concerns. Reference intervals could also falsely exclude otherwise eligible volunteers making the process of trial recruitment and enrollment challenging. Likewise, inappropriate participant selection may bias trial results such that data become less generalizable to the healthy population being studied.

Prior to 2008, in most large-population based reference interval studies, the health status of participants were not assessed thoroughly and no additional medical or laboratory examinations were performed [3,4]. Majority of the reports also focused only on adults [3,4,8,9,10] and few included biochemistry reference values [5,6,7,11]. With publication of the International Federation of Clinical Chemistry (IFCC) and Clinical and Laboratory Standards Institute (CLSI) revised guidelines in 2008 [12], reference interval studies in Africa have increased [13,14,15,16,17] with some including children in their analyses [18,19]. These studies demonstrate differences in reference values compared to established US reference intervals [5,7,13,14,16,19,20].

Here, we present hematological and biochemical indices of healthy volunteers aged 6–32 years old who participated in the Phase Ib clinical trial. From the screening of healthy volunteers, we had the opportunity to collect and compare both hematological and biochemical data from these volunteers and re-assess previously reported reference intervals in Uganda as well as in Africa. Besides, the well-defined inclusion and exclusion criteria applied in the trial represent a data set from a northern Uganda population that can typically participate in intervention trials and vaccine studies. The results also underscore the need for region-specific children reference intervals that can be more appropriate for the population.

## 2. Experimental Section

### 2.1. Study Population

In this study, only the screening visit data of volunteers who participated in the BK-SE36 Phase Ib clinical trial [2] was included in the analysis. The study took place at Lira Medical Center in Lira district, Northern Uganda. Lira district is approximately 1063 m above sea level and lies 347 km north of Kampala, the capital of Uganda. The district is predominantly agricultural, with the population comprising largely small landholder subsistence farmers: growing beans and cassava; owning chicken and goats [21]. The staple foods are mainly maize, cassava, sorghum and sweet potatoes. The area has a tropical climate with a temperature range of 17.4–30.3 °C and an average rainfall of 1478 mm/year [21].

Briefly, there were two stages of screening. Stage 1 was in 21–40 year-olds (n = 56) and Stage 2 was in 3 age cohorts (6–10, 11–15 and 16–20 years old). Screening for Stage 1 was conducted in April–May 2010 and for Stage 2 in September–October 2010. The trial was conducted in compliance with the study protocol, the International Conference on Harmonization’s Good Clinical Practice standards, the Declaration of Helsinki and Uganda regulatory requirements. Approvals for the protocol, subject information and informed consent forms were obtained from the ethical institutional review committees (IRC) of Osaka University (RIMD-IRC) (Japan), Research Foundation for Microbial Diseases of Osaka University (BIKEN-IRC) (Japan), Med Biotech Laboratories (MBL-IRC) (Uganda), and regulatory bodies Uganda National Council for Science and Technology (UNCST) and the Uganda National Drug Authority (NDA). Informed consent was obtained in English, Swahili or Luo (the language commonly spoken locally). Written consent was obtained from all enrolled subjects, in addition to individual assent from children aged eight and above. All participants were screened through a medical history questionnaire and complete physical examination. Females of child bearing age were tested for pregnancy. Selected participants were healthy, with no obvious symptoms/signs of either acute or chronic respiratory, cardiovascular, gastrointestinal, hepatic or renal disease; no history of blood donation/transfusion within one month and, for females, non-lactating. A detailed report of the study population recruitment, enrollment, inclusion/exclusion criteria, and blood sampling has been published [2].

Whole blood samples were collected from fasting subjects via venipuncture with a Vacutainer system (Becton Dickinson Biosciences, Franklin Lakes, NJ, USA), with all blood drawn in the morning (7:00–12:00 am) and hematological and biochemistry tests performed within the day. The samples were also checked for malaria parasites. Blood sampling and processing were done according to standard operating procedures by trained laboratory staff (EDTA treated tubes for hematology and untreated tubes for biochemistry tests). Hematological indices were measured by Sysmex KX-21N (Sysmex Corporation, Kobe, Japan); and biochemical indices by Cobas C111 (Roche Diagnostics, Mannheim, Germany) per manufacturer’s instructions. Daily quality control was performed on the equipment and all laboratory procedures adhered to Good Clinical Laboratory Practices. Calibrators and controls were obtained from the instrument manufacturer. For hematology tests, daily running of the 3-part Differential Whole Blood Para^®^ Check (Para^®^ Check Low, Para^®^ Check Normal and Para^®^ Check High) controls ensured calibration and quality control limits for each analyte. For Cobas C111, we used normal (PreciNorm U) and abnormal (PreciPath U) control sera from Roche Diagnostics. In addition, the laboratory was under an external quality assurance program with the South African National Accreditation System. Assessments included white blood cell (WBC) count, red blood cell (RBC) count, hemoglobin (Hb), hematocrit (Hct), platelet count, alkaline phosphatase (AL-P), alanine aminotransferase (ALT), aspartate aminotransferase (AST), γ-glutamyl-transferase (γ-GTP), amylase, albumin, total protein, total bilirubin, cholesterol, glucose, creatinine, urea nitrogen (BUN), uric acid, potassium and sodium.

### 2.2. Statistical Methods

Data were pooled, analyzed untransformed and are shown as mean, median, maximum-minimum values, and 95% reference intervals according to CLSI C28-A3 guideline [12]. Of the measured analyte, 70% did not conform to a Gaussian distribution according to the D’Agostino and Pearson and Shapiro-Wilk tests for normality. Outlier exclusion was the recommended Dixon-Reed’s outlier method. Non-parametric percentiles and 90% CIs were determined. Possible partitioning to two broad age groups: 6–15 years-old and 16–32 years-old were done using the robust method with possible outliers detected by visual inspection of distributions and Tukey’s criterion. Analytes that have significant age differences based on the non-parametric Mann-Whitney U test are shown in Table 3. Statistical analyses were performed using MedCalc for Windows, version 13.1.1.0 (MedCalc Software, Ostend, Belgium) and GraphPad Prism 6 (GraphPad Software; La Jolla, CA, USA).

## 3. Results and Discussion

### 3.1. Baseline Characteristics of the Study Population

From April–May and September–October 2010 (two sampling periods), we enrolled a total of 140 healthy individuals [2]. We report on the health status of these individuals based on hematological and biochemical data obtained. The final analysis cohort pooled participants from age 6–32 years-old. Table 1 shows the general characteristics of the study population. In adults (21–32 years), mean age and body mass index (BMI) were similar across gender. Lower mean diastolic and systolic blood pressure (BP) but higher mean pulse rate was obtained from female than male volunteers. In younger cohorts, as expected weight, height and BMI increased with age. Diastolic and systolic BP were similar across age groups, although pulse rate of 6–10 years and 11–15 year-old cohorts were significantly higher than 16–20 years cohort. In the 16–20 year-old cohort, male-to-female ratio was 1:1. However, there was a preponderance of females in the 6–10 year-old cohort (representing 54% against 46%) and in 11–15 year-old cohort (82% *vs.* 18% males).

### 3.2. Hematological Analysis

The mean, standard deviation, median, minimum/maximum values and 95% reference intervals for hematology analytes are given in Table 2. Volunteers had low WBC and platelet counts. Ranges for RBC count, hematocrit and hemoglobin values were generally broad when compared to Western reference intervals obtained from Pathology Associates Medical Laboratories [22]. The low hemoglobin; hematocrit; white blood cell, erythrocyte and platelet counts in Africans compared to northern European population or whites have been shown to be related to ethnicity and/or genetic factors [23,24,25]. Significant age differences for red cell indices were obtained when reference intervals were partitioned to two broad age groups (6–15 and 16–32 years-old). When computed by the robust method as recommended by CLSI C28-A3 guidelines, age differences were evident in the upper values for RBC count (*p* = 0.009), hemoglobin (*p* < 0.0001) and hematocrit (*p* < 0.0001) in 16–32 year-old compared to 6–15 year-old (Table 3). The increase in hemoglobin, hematocrit and erythrocyte counts; as well as the decrease in platelet counts according to age has been reported [4,19,20]. Values were within the 2.5 and 97.5 percentiles obtained from Uganda and other African countries with an altitude below 2000 m (Table 4). Higher hemoglobin and hematocrit values were reported for Ethiopia (at >2300 m above sea level) and lower hemoglobin and hematocrit were noted for Ghana (at 60–150 m above sea level).

### 3.3. Biochemical Analysis

The mean, standard deviation, median, minimum/maximum values and 95% reference intervals for biochemistry values are summarized in Table 2. Most biochemical analytes were within reference intervals reported for the Western population [22,26] with the exception of ALT, γ-GTP, creatinine, protein, bilirubin, potassium and sodium. The lower limit for ALT; higher limits for protein, bilirubin and sodium have been recorded in the Ugandan population [7]. We also observed significant age differences in reference intervals for the following indices: AL-P (*p* < 0.0001), AST (*p* < 0.0001), amylase (*p* = 0.02), glucose (*p* < 0.0001), creatinine (*p* < 0.0001), urea nitrogen (BUN, *p* = 0.002), uric acid (*p* < 0.0001), potassium (*p* = 0.002) and sodium (*p* < 0.0001) (Table 3). Using the robust method, in adults, the reference ranges obtained fell at the lower end of the current reference intervals reported for Uganda and other African countries (Table 5). In adolescents and young children there are, at present, only a handful of studies available (Table 6).

**Table 1:** Study population characteristics in different age groups (n = 140).

Analyte	6–10 Years old (n = 28)			11–15 Years old (n = 28)			16–20 Years old (n = 28)			Male >20 Years old (n = 28)			Female >20 Years old (n = 28)		
	Mean	SD	Min/Max	Mean	SD	Min/Max	Mean	SD	Min/Max	Mean	SD	Min/Max	Mean	SD	Min/Max
Age *	8.11	1.52	6/10	12.68	1.25	11/15	17.96	0.99	16/20	22.61	1.26	21/25	22.14	2.14	21/32
BMI	16.06	1.54	13.9/19.8	18.25	2.38	15.4/24.2	20.60	1.90	18.1/25	21.35	1.82	18/25	22.14	2.56	18.4/28
Weight	28.35	5.61	17/42	43.18	8.00	33.5/63	57.79	4.27	49/67	62.78	6.36	50.1/75.3	59.74	7.53	48.5/74.5
Height	132.2	9.99	110.5/153	153.5	7.49	140/169.7	167.8	7.13	157/182.2	171.4	5.90	161.5/184.3	164.2	5.87	153.7/183
**Vital signs**															
DBP	69.61	4.49	60/78	71.29	6.92	60/82	73.43	6.55	60/86	73.54	7.10	58/87	72.64	7.37	57/87
SBP	115.6	8.19	96/126	119.0	8.12	105/129	118.9	8.05	102/131	120.1	13.46	100/142	108.8	11.07	89/135
Pulse rate	82.82	10.23	66/107	78.79	10.57	60/97	69.64	8.73	55/90	67.25	6.26	55/79	70.25	8.96	55/92

* Age (year); BMI (kg/m^2^); weight (kg); height (cm); DBP (diastolic blood pressure; mmHg); SBP (systolic blood pressure, mmHg); pulse rate (bpm).

**Table 2:** Means, median and 95% percentile reference ranges for hematology and biochemistry analytes in 6–32 year old.

Analyte	n	Mean	SD	Median	Min/Max	95% Reference Interval			
						Lower Limit	90% CI	Upper Limit	90% CI
WBC ^§^	140	5.66	1.47	5.4	3.3/10.8	3.6	3.3–3.8	9.99	8.3–10.8
RBC	140	5.06	0.55	4.98	3.9/6.6	4.1	3.9–4.2	6.28	6.11–6.58
Hb	140	13.75	1.64	13.5	10/18.2	10.55	10–11.6	17.5	16.6–18.2
Hct	140	41.11	4.35	40.8	31.4/51.5	33.3	31.4–35.5	50.35	49.4–51.5
Platelet	140	228.5	63.23	222	115/473	118.53	115–135	392.7	337–473
AL-P	138	151.8	106.9	95.8	42.8/456.2	44.1	42.8–48.2	394.9	355.8–456.2
ALT	140	15.99	5.61	14.95	6.5/43.5	8.9	6.5–9.7	35.31	24.4–43.5
AST	139	21.53	4.37	21.3	13.4/36.5	14.3	13.4–15.5	33.9	28.7–36.5
γ-GTP	139	13.09	6.52	11.6	3.5/45.6	4.05	3.5–5.5	29.85	26–45.6
Amylase	140	27.21	8.95	25.65	12.7/74.9	15.17	12.7–16.3	52.75	43.4–74.9
Albumin	140	4.6	0.26	4.61	3.8/5.3	4.08	3.8–4.2	5.04	4.99–5.3
Protein	140	7.6	0.59	7.5	5.7/9.5	6.25	5.7–6.7	8.6	8.5–9.5
Bilirubin	140	0.54	0.38	0.4	0.1/1.9	0.1	0.1–0.1	1.7	1.3–1.9
Cholesterol	140	128.7	25.1	128.1	71.6/188.9	85.55	71.6–91.8	174.15	167.5–188.98
Glucose	140	84.7	10.23	82.78	67.6/118.96	69.5	67.6–71.8	114.6	103.9–118.96
Creatinine	140	0.59	0.15	0.6	0.4/1.0	0.4	0.4–0.4	0.95	0.9–1.0
BUN	140	14.94	4.55	14.16	2.84/29.68	8.29	2.84–9.14	24.9	23.2–29.68
Uric acid	140	3.85	0.83	3.8	2.1/6.9	2.4	2.1–2.6	5.59	5.3–6.9
Potassium	140	4.24	0.39	4.24	3.4/5.42	3.57	3.41–3.64	5.27	4.9–5.42
Sodium	140	139.6	3.2	139.15	135.3/149.4	135.5	135.3–135.8	148.5	145.8–149.4

^§^ WBC (leukocyte count, ×10^9^/L); RBC (erythrocyte count, ×10^12^/L); Hb (hemoglobin, g/dL); Hct (hematocrit, %); Platelet count (×10^9^/L); AL-P (alkaline phosphatase, U/L); ALT (alanine aminotransferase, U/L); AST (aspartate aminotransferase, U/L); γ-GTP (γ-glutamyl transpeptidase, U/L); Amylase (serum amylase, U/L); Albumin (g/dL); Protein (total protein, g/dL); Bilirubin (total bilirubin, mg/dL); Cholesterol (total cholesterol, mg/dL); Glucose (mg/dL); Creatinine (mg/dL); BUN (urea nitrogen, mg/dL); Uric acid (mg/dL); Potassium (mmol/L); Sodium (mmol/L); SD = standard deviation; CI = confidence interval.

**Table 3:** 95% Reference interval for hematology and biochemistry analytes by age group *.

Analyte	6–15 Years old					16–32 Years old				
	n	Lower Limit	90% CI	Upper Limit	90% CI	n	Lower Limit	90% CI	Upper Limit	90% CI
**Hematology**										
RBC ^§^	56	4.1	3.9–4.2	5.7	5.55–5.84	84	3.9	3.7–4.1	6.4	6.16–6.55
Hb	56	11.2	10.9–11.5	14.5	14.2–14.8	84	10.9	10.3–11.4	17.9	17.3–18.4
Hct	55	34.3	33.66–35	42.8	41.9–43.6	84	33.7	32.5–35.2	52.1	50.8–53.3
**Biochemistry**										
AL–P	53	111.1	82.8–140.8	419.7	385.1–450.1	78	31.9	26.7–37.47	108.4	100.5–114.5
AST	55	16.14	14.96–17.5	29.9	28.7–31.1	80	12.99	11.99–14.1	26.11	25–27.15
Amylase	55	12.42	10.2–15.1	36.3	34.16–38.6	76	14.43	12.6–16.4	37.3	35–39.3
Glucose	54	66.4	62.7–70.65	109.3	103.9–114	80	67.2	65.4–69.3	91.3	89.1–93.5
Creatinine	55	0.31	NC	0.61	NC	84	0.43	NC	0.92	NC
BUN	56	5.14	3.7–6.6	21.29	19.4–22.8	83	6.05	4.69–7.4	24.4	22.5–25.9
Uric acid	56	2	1.78–2.25	4.88	4.58–5.18	83	2.5	2.28–2.77	5.59	5.34–5.82
Potassium	52	3.46	3.34–3.57	4.65	4.54–4.74	84	3.58	3.47–3.69	5.02	4.91–5.12
Sodium	56	134.2	133.5–134.8	141	140.2–141.7	84	133.87	133–134.8	147.4	146.2–148.54

* Calculated by the robust method recommended by CLSI C28-A3 guideline. NC, not calculated (not possible to calculate) for the subgroup. ^§^ RBC (erythrocyte count, ×10^12^/L); Hb (hemoglobin, g/dL); Hct (hematocrit, %); AL-P (alkaline phosphatase, U/L); AST (aspartate aminotransferase, U/L); Amylase (serum amylase, U/L); Glucose (mg/dL); Creatinine (mg/dL); BUN (urea nitrogen, mg/dL); Uric acid (mg/dL); Potassium (mmol/L); Sodium (mmol/L).

**Table 4:** Comparison of hematology ranges with other studies in Africa: 16–32 years-old.

Analy te	This study* (16–32 years old)	Kenya, Uganda, Zambia, Rwanda ^1^ (18–60 years old)	Uganda ^2^ (18–56 years old)	Western Kenya ^3^ (18–34 years old)	Kericho–Kenya ^4^ (18–55 years old)	Tanzania ^5^ (19–48 years old)	Ethiopia ^6^ (15–45 years old)	Central Africa ^7^ (17–58 years old)	Togo ^8^ (17–58 years old)	Ghana ^9^ (18–59 years old)	South Africa ^10^ (18–55 years old)
RBC ^§^	3.9–6.4	3.8–6.2 ^†^	3.5–6.1 ^†^	4.3–6.5 ^†^ 3.4–5.7	4–6.2 ^†^	4.01–6.12 ^†^	4.3–5.9 3.7–5.2	4.50–6.10 3.42–5.44	3.1–6.4 ^†^	3.39–5.83 ^†^	4.19–5.85 ^†^ 3.93–5.40
n	84	2105	664	160	1541	272	485	150	1349	623	678
Hb	10.9–17.9	10.5–17.5 ^†^	10.8–17.1 ^†^	11.4–16.9 ^†^ 8.0–14.2	6.7–11.1 ^†^	11.7–17.2 ^†^	13.9–18.3 12.2–16.6	12.3–17.3 9.1–14.9	10–18.4 ^†^	9.8–16.0 ^†^	13.4–17.5 ^†^ 11.6–16.4
n	84	2105	664	160	1541	276	485	150	1349	624	678
Hct	33.7–52.1	31.6–51.7 ^†^	31.2–49.5 ^†^	32.6–51.5 ^†^ 23.2–44.3	30–50 ^†^	36.5–52.7 ^†^	41.6–55.1 35.3–48.8	39–52 28–44	28–54 ^†^	28.9–48.7 ^†^	39–51 ^†^ 34–48
n	84	2105	664	160	1541	276	485	150	1349	625	678

^§^ RBC (erythrocyte count, ×10^12^/L); Hb (hemoglobin, g/dL); Hct (hematocrit, %). ^1^ Karita *et al*., 2009 [13] and IAVI, 2008 [27]; ^2^ Eller *et al*., 2008 [7]; ^3^ Zeh *et al*., 2011 [19]; ^4^ Kibaya *et al*., 2008 [6]; ^5^ Saathoff *et al*., 2008 [5]; ^6^ Tsegaye *et al*., 1999 [9]; ^7^ Menard *et al*., 2003 [10]; ^8^ Kueviakoe *et al*., 2011 [15]; ^9^ Dosoo *et al*., 2012 [16]; ^10^ Lawrie *et al*., 2009 [14]. Shown are 95% reference intervals. * Calculated by the robust method recommended by CLSI C28-A3 guideline. ^†^ Indicates analytes with significant gender differences. If two values are shown, only separate male and female reference intervals, respectively were reported.

**Table 5:** Comparison of biochemistry ranges with other studies in Africa: 16–32 years-old.

Analyte	This study (21–32 years old)	Kenya, Uganda, Zambia, Rwanda ^1^ (18–60 years old)	Uganda ^2^ (18–56 years old)	Western Kenya ^3^ (18–34 years old)	Kericho–Kenya ^4^ (18–55 years old)	Tanzania ^5^ (19–48 years old)	Ghana ^6^ (18–59 years old)
**Enzymes**							
AL–P (U/L) ^§^	31.9–108.4	48–164	44–151			45.6–158.4	85–241 ^†^
n	78	2103	433			236	595
AST (U/L)	12.99–26.11	14–60	12.3–34.8 ^†^	13.8–50.4	13.8–42.3 ^†^	14.3–48.1 ^†^	14–51 ^†^
n	80	2103	845	293	1533	269	588
Amylase (U/L)	14.43–37.3	35–159	45.6–173.6		38.3–163.0 ^†^	42.8–164.4	32–139 ^†^
n	76	2103	433		1205	270	621
**Metabolism**							
Glucose (mg/dL)	67.2–91.3			37.84–118.92	55.8–102.7	52.61–94.23	64.87–115.32
n	80			293	1541	262	622
**Kidney function**							
Creatinine (mg/dL)	0.43–0.92	0.53–1.23	0.5–1.2 ^†^	0.57–1.28	0.62–1.15	0.47–1.02 ^†^	0.55–1.33 ^†^
n	84	2103	851	293	1540	270	615
BUN (mg/dL)	6.05–24.4		4.6–15.5 ^†^	3.36–14.29	3.92–12.89 ^†^	4.29–13.89 ^†^	2.52–15.97 ^†^
n	83		848	293	1541	265	622
Uric acid (mg/dL)	2.5–5.59		3.3–7.8 ^†^			2.76–7.5 ^†^	1.52–6.7 ^†^
n	83		729			270	620
**Electrolytes**							
Potassium(mmol/L)	3.58–5.02		3.4–4.8		3.9–5.8 ^†^	3.83–5.52 ^†^	3.6–5.2 ^†^
n	84		849		1535	269	583
Sodium (mmol/L)	133.9–147.4		135.3–147.6 ^†^		141.4–152.5	133.6–142.5 ^†^	135–150
n	84		849		1541	257	541

^§^ AL-P, alkaline phosphatase; AST, aspartate aminotransferase; BUN, urea nitrogen. ^1^ Karita, *et al*., 2009 [13] and IAVI, 2008 [27]; ^2^ Eller, *et al*., 2008 [7]; ^3^ Zeh, *et al*., 2011 [19]; ^4^ Kibaya, *et al*., 2008 [6]; ^5^ Saathoff, *et al*., 2008 [5]; ^6^ Dosoo, *et al*., 2012 [16]. Shown are 95% reference intervals. When a value is preceeded by ^†^, analyte was reported to have significant gender differences (*p* < 0.05).

**Table 6:** Comparison of hematology and biochemistry ranges with other studies in Africa: 6–15 years-old.

Analyte	This study *	Uganda ^1^ (90% intervals)	Western Kenya ^2^
**Hematology**			
RBC (×10^12^/L) ^§^	4.08–5.7	3.8–5.4 ^†^	(4.1–5.8) (3.3–5.4) ^†^
n	56	731	133
Hb (g/dL)	11.18–14.55	10.0–13.7 ^†^	(10.6–15.6) (8.1–14.2) ^†^
n	56	731	133
Hct (%)	34.3–42.8	29.2–39.4 ^†^	(29.3–48.1) (24.8–43.1) ^†^
n	55	731	133
**Biochemistry**			
AL-P (U/L) ^§^	111.1–419.7		
n	53		
AST (U/L)	16.14–29.9		(17.0–59.2) (12.0–43.1) ^†^
n	55		293
Amylase (U/L)	12.42–36.3		
n	55		
**Metabolism**			
Glucose (mg/dL)	66.4–109.3		(37.84–118.92)
n	54		293
**Kidney function**			
Creatinine (mg/dL)	0.31–0.61		
n	55		
BUN (mg/dL)	5.14–21.29		(3.36–14.29)
n	56		293
Uric acid (mg/dL)	2–4.88		
n	56		
**Electrolytes**			
Potassium (mmol/L)	3.46–4.65		
n	52		
Sodium (mmol/L)	134.2–141		
n	56		

^§^ RBC, erythrocyte count; Hb, hemoglobin; Hct, hematocrit; AL-P, alkaline phosphatase; AST, aspartate aminotransferase; BUN, urea nitrogen. ^1^ Except for Lugada *et al*., 2004 [4], shown are 95% reference intervals. When two values are provided, these correspond to male and female ranges, respectively. ^†^ Indicates analytes with significant age and gender differences (*p* < 0.05); age range listed is 6–12 years for ^1^ Lugada *et al*., 2004 [4]; 13–17 years for ^2^ Zeh *et al*., 2011 [19].

## 4. Conclusions

The opportunity to re-assess currently used reference intervals in northern Uganda presented itself when we conducted a phase 1b trial in Lira, Uganda. Reference intervals serve as a valuable guide for interpretation of laboratory results. Hematology and biochemistry analytes are influenced by a number of factors such as sex, age, ethnic origin, environment (including geographical location, access to health care), constitutional abnormalities (thalassemia, sickle cell disease), pathologic conditions (malaria, HIV and HBV viral infections); and to some extent demographic changes and technological advancement in analyte measurements [4,5,6,7,13,14,15,16,17,18,19,20,23,27,28].

The revised international guidelines from CLSI recommend that if it is not possible to establish detailed reference studies from at least 120 individuals, validation using as few as 20 samples could be helpful together with comparison using published reference intervals and performing *posteriori* methods [29,30]. We, thus, examined and compared the variability of hematological and biochemical analytes obtained during participant screening for trial enrollment. The analytes were commonly used for clinical assessments to guide inclusion/exclusion into the trial and for adverse event evaluation.

The studied population is heterogeneous with regards to age and gender. However, overall, the upper and lower limits of the reference intervals obtained in this study were almost the same as the limits reported in previous adult hematologic reference intervals (Table 4). Trends of age-specific differences in some parameters were also evident although this needs further verification in larger cohorts (Table 6). At present there are few reports available for adolescents and younger age groups, especially on biochemistry analytes in Africa [11,19,31,32].

Whether gender or age would alter the reference intervals obtained in the present study is for further studies. The screening activity did not screen out all medical conditions such as hepatitis B, HIV or other parasitic infections except for malaria. We have no information on social habits (tobacco use, diet, alcohol consumption, exercise, *etc.*) as these were not obtained during enrollment of the volunteers (e.g., factors such as smoking has been reported to affect hemoglobin values [14]). We did, however, have a well-defined trial inclusion and exclusion criteria, and all subject were evaluated for both hematological and biochemical indices. Volunteers were encouraged to come for screening to the study clinic after fasting for at least 8 h. Breakfast was given to all participants afterwards and this assisted in a high level of compliance with this request, although in some cases fasting was interpreted as an instruction not to eat or drink and, thus, some volunteers were mildly dehydrated during blood sample collection. Nevertheless, the study provides preliminary data needed to facilitate health care and research trials in a population in which reference interval generation has not yet been done. Participants in the study also represent volunteers that would participate in clinical trials. Importantly, we also highlight the need for local data especially in younger age groups as their participation in clinical trial is solicited.
